# Comparison of ^3^He and ^129^Xe MRI for evaluation of lung microstructure and ventilation at 1.5T

**DOI:** 10.1002/jmri.25992

**Published:** 2018-03-05

**Authors:** Neil J. Stewart, Ho‐Fung Chan, Paul J.C. Hughes, Felix C. Horn, Graham Norquay, Madhwesha Rao, Denise P. Yates, Rob H. Ireland, Matthew Q. Hatton, Bilal A. Tahir, Paul Ford, Andrew J. Swift, Rod Lawson, Helen Marshall, Guilhem J. Collier, Jim M. Wild

**Affiliations:** ^1^ Academic Unit of Radiology University of Sheffield Sheffield UK; ^2^ Novartis Institutes for Biomedical Research Cambridge Massachusetts USA; ^3^ Academic Unit of Clinical Oncology University of Sheffield Sheffield UK; ^4^ Sheffield Teaching Hospitals NHS Foundation Trust Sheffield UK

**Keywords:** hyperpolarized gas, lung MRI, xenon‐129, helium‐3, chronic obstructive pulmonary disease, lung cancer, repeatability

## Abstract

**Background:**

To support translational lung MRI research with hyperpolarized ^129^Xe gas, comprehensive evaluation of derived quantitative lung function measures against established measures from ^3^He MRI is required. Few comparative studies have been performed to date, only at 3T, and multisession repeatability of ^129^Xe functional metrics have not been reported.

**Purpose/Hypothesis:**

To compare hyperpolarized ^129^Xe and ^3^He MRI‐derived quantitative metrics of lung ventilation and microstructure, and their repeatability, at 1.5T.

**Study Type:**

Retrospective.

**Population:**

Fourteen healthy nonsmokers (HN), five exsmokers (ES), five patients with chronic obstructive pulmonary disease (COPD), and 16 patients with nonsmall‐cell lung cancer (NSCLC).

**Field Strength/Sequence:**

1.5T. NSCLC, COPD patients and selected HN subjects underwent 3D balanced steady‐state free‐precession lung ventilation MRI using both ^3^He and ^129^Xe. Selected HN, all ES, and COPD patients underwent 2D multislice spoiled gradient‐echo diffusion‐weighted lung MRI using both hyperpolarized gas nuclei.

**Assessment:**

Ventilated volume percentages (VV%) and mean apparent diffusion coefficients (ADC) were derived from imaging. COPD patients performed the whole MR protocol in four separate scan sessions to assess repeatability. Same‐day pulmonary function tests were performed.

**Statistical Tests:**

Intermetric correlations: Spearman's coefficient. Intergroup/internuclei differences: analysis of variance / Wilcoxon's signed rank. Repeatability: coefficient of variation (CV), intraclass correlation (ICC) coefficient.

**Results:**

A significant positive correlation between ^3^He and ^129^Xe VV% was observed (*r* = 0.860, *P* < 0.001). VV% was larger for ^3^He than ^129^Xe (*P* = 0.001); average bias, 8.79%. A strong correlation between mean ^3^He and ^129^Xe ADC was obtained (*r* = 0.922, *P* < 0.001). MR parameters exhibited good correlations with pulmonary function tests. In COPD patients, mean CV of ^3^He and ^129^Xe VV% was 4.08% and 13.01%, respectively, with ICC coefficients of 0.541 (*P* = 0.061) and 0.458 (*P* = 0.095). Mean ^3^He and ^129^Xe ADC values were highly repeatable (mean CV: 2.98%, 2.77%, respectively; ICC: 0.995, *P* < 0.001; 0.936, *P* < 0.001).

**Data Conclusion:**

^129^Xe lung MRI provides near‐equivalent information to ^3^He for quantitative lung ventilation and microstructural MRI at 1.5T.

**Level of Evidence**: 3

**Technical Efficacy** Stage 2

J. Magn. Reson. Imaging 2018;48:632–642.

THE MAJORITY of hyperpolarized gas lung magnetic resonance imaging (MRI) studies that have been performed to date in patients with lung diseases have used ^3^He gas, exploiting its intrinsically stronger MRI signal when compared to ^129^Xe. However, recent years have seen an increase in ^129^Xe lung MRI^1^ studies in patients for assessment of lung ventilation,^2^, ^3^, ^4^, ^5^ microstructure,^6^, ^7^ and regional gas exchange^8^ with this naturally abundant isotope. This renewed interest is attributable to the relative scarcity of ^3^He gas,^9^ and parallel developments in ^129^Xe gas polarization technology^10^, ^11^, ^12^, ^13^ and MRI pulse sequences^14^ that have helped bridge the gap in image quality due to the difference in gyromagnetic ratio between the two nuclei.

Previous comparisons of the sensitivity of ^3^He and ^129^Xe MRI for assessment of lung ventilation and microstructure have been reported in healthy subjects, former smokers,^7^ patients with chronic obstructive pulmonary disease (COPD),^3^, ^15^ and asthma.^16^ Generally, lung ventilation and microstructural information of similar diagnostic quality has been obtained with the two nuclei, despite their different diffusivity in the lung airspaces. However, all of these reported studies were carried out at 3T, at relatively modest spatial resolution (15 mm and 30 mm slice thickness for ventilation and diffusion‐weighted microstructural MRI, respectively), and at a single site.^3^, ^7^, ^15^, ^16^ Magnetic susceptibility differences at 1.5T and 3T have been shown to have some impact on lung ventilation and diffusion‐weighted microstructural MRI with both gases^17^, ^18^; however, quantitative information derived from ^3^He and ^129^Xe techniques at the most‐reported field strength to date for lung MRI (1.5T) has yet to be systematically compared. This information is critical for multisite implementation and widespread clinical dissemination of ^129^Xe MRI in the future.

In addition, future clinical use of ^129^Xe will require quantitative data regarding the repeatability and robustness of associated MRI biomarkers of lung disease. The most commonly reported quantitative metrics of lung ventilation from conventional, static hyperpolarized gas images are percentage ventilated volume (VV%) and ventilation defect percentage (VDP = 100% – VV%). Same‐day ^3^He VDP measurements were reported to be highly repeatable at 3T in patients with COPD,^19^, ^20^ while 1‐week repeatability was relatively poorer. Good same‐day and same‐week repeatability of ^3^He VV% has been reported in cystic fibrosis (CF) patients at 1.5T^21^ and 3T,^22^ respectively. One report of high same‐session repeatability of ^129^Xe VDP in healthy subjects and asthma patients has been published recently^23^; however, multisession, multiday variability in quantitative lung ventilation metrics derived from ^129^Xe MRI has not been assessed. Delivery of several doses of ^129^Xe gas for ventilation imaging on the same/subsequent day to subjects with a variety of pulmonary conditions has been reported.^2^, ^24^ However, the primary purpose of those studies was to assess safety and tolerability, and ventilation volume repeatability was not evaluated.

The global mean ^3^He apparent diffusion coefficient (ADC) in the lung airspaces derived from diffusion‐weighted MRI is a well‐established marker of alveolar microstructural change. Comprehensive investigations of same‐session^25^ and multiday^26^ repeatability of mean ^3^He ADC values have demonstrated a high intra‐individual repeatability in healthy subjects and patients with emphysema. Furthermore, studies at 3T have highlighted a higher repeatability of ^3^He ADC when compared to ^3^He VDP in patients with COPD.^19^, ^20^ However, to our knowledge, interscan repeatability of ^129^Xe ADC has not been assessed to date. Evaluation of the repeatability of lung ventilation and microstructural metrics derived from ^129^Xe MRI against ^3^He MRI and pulmonary functional tests is therefore necessary to facilitate the clinical dissemination of these methods.

Taking the gaps in the literature discussed above into account, the purpose of this work was to evaluate the applicability and repeatability of quantitative metrics of ^129^Xe ventilation and diffusion‐weighted MRI as compared to the equivalent ^3^He measurements made at a field strength of 1.5T.

## Materials and Methods

### Subjects

We present an analysis of MR data obtained from several distinct studies. Two groups of healthy nonsmokers: a) 11 participants (age = 43 ± 6 years), and b) three participants (age = 31 ± 4 years), all with no history of smoking or respiratory disorders; five ex‐smokers with a pack history of ≥10 years and no history of respiratory disorders (age = 51 ± 2 years); five patients with COPD (GOLD stage: 3D [*n* = 4], 3B [*n* = 1]; age = 67 ± 7 years); and 16 patients with nonsmall‐cell lung cancer (NSCLC) (age = 67 ± 12 years) were recruited for separate studies approved by the National Research Ethics Committee, with governance approval from the local National Health Service research committee. All subjects provided written informed consent. Patient demographics and pulmonary function test (PFT) results are summarized in Table 1. Subjects with a resting oxygen saturation of <90%, unstable cardiac disease, or (for NSCLC patients) comorbid conditions that precluded radiotherapy, were excluded.

**Table 1:** Summary of Subject Demographics, PFT Results, and ^3^He and ^129^Xe MRI Parameters

	Subject group				
	Healthy nonsmokers				
Parameter	a	b	Healthy ex‐smokers	NSCLC patients	COPD patients
Age (yrs) (# of Subjects)	43.0 ± 6.4 (6M, 5F)	30.7 ± 3.5 (3M)	51.0 ± 2.3 (3M, 2F)	66.9 ± 12.0 (10M, 6F)	67.4 ± 6.5 (2M, 3F)
FEV_1_ (%‐pred)	101.0 ± 12.5	92.6 ± 15.9	94.9 ± 9.8	72.7 ± 25.0	38.0 ± 6.6
FEV_1_/FVC (%)	77.1 ± 7.2	77.0 ± 7.6	75.1 ± 13.2	57.1 ± 16.5	29.7 ± 5.9
D_LCO_ (%‐pred)	89.6 ± 17.8	102.3 ± 9.3	103.5 ± 16.2	56.6 ± 31.0	42.5 ± 32.4
^3^He VV%	—	98.4 ± 0.60	—	79.6 ± 11.7	71.4 ± 7.8
^129^Xe VV%	—	96.6 ± 0.32	—	70.8 ± 13.3	59.8 ± 10.4
^3^He ADC_glob_ (cm^2^.s^−1^)	0.190 ± 0.017	—	0.211 ± 0.022	—	0.432 ± 0.127
^129^Xe ADC_glob_ (cm^2^.s^−1^)	0.038 ± 0.003	—	0.043 ± 0.004	—	0.073 ± 0.019

FEV_1_ = forced expiratory volume in 1 second; FVC = forced vital capacity; D_LCO_ = diffusing capacity of the lung for carbon monoxide; %‐pred = PFTs expressed as a percentage of a predicted value, based on the subject's age, height, and other demographic factors.

### MRI Technique

All subjects underwent MRI at 1.5T (GE HDx, GE Healthcare, Milwaukee, WI). Flexible quadrature radiofrequency coils were employed for transmission and reception of MR signals at the Larmor frequencies of ^3^He and ^129^Xe (Clinical MR Solutions, Brookfield, WI). ^3^He and ^129^Xe gas was polarized by collisional spin‐exchange optical pumping, using a prototype commercial ^3^He polarizer (MITI, Durham, NC) (average polarization ∼25%) and a home‐built ^129^Xe polarizer^12^, ^27^ (average polarization ∼10–15%), respectively.

**Figure 1:**
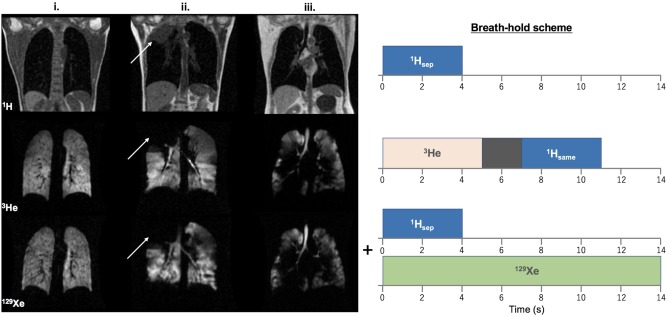
Left: Comparison of ^3^He and ^129^Xe MR ventilation images of i. a healthy nonsmoker (group b), ii. a patient with NSCLC (white arrows indicate the location of a lesion), and iii. a patient with COPD. (Note: The slice thickness of ^3^He images is half that of ^129^Xe images; see Table 2). Right: Corresponding breath‐hold scheme for ventilation imaging scans. Scans were acquired in the order shown, with a change of RF coil and repositioning of the patient between ^3^He and ^129^Xe scans.

Patients with NSCLC and COPD, and three healthy nonsmokers (group b) underwent 3D lung ventilation MRI with a steady‐state free precession (SSFP) sequence at breath‐hold after inhalation of ^3^He or ^129^Xe gas.^14^, ^28^ ^1^H images of the thorax were also acquired for anatomical reference and calculation of total lung volume. As illustrated in Fig. 1 (right), ^1^H images were acquired in both the same breath‐hold^29^ and a separate breath‐hold as ^3^He ventilation images (same breath‐hold for ease of image registration; separate breath‐hold in case same breath‐hold acquisition failed or could not be registered). Due to the length of the ^129^Xe ventilation imaging breath‐hold, ^1^H images were acquired in a separate breath‐hold. VV% was derived by segmentation (and registration if required) of paired sets of hyperpolarized gas and corresponding ^1^H images as described previously^28^ (VV% = volume of "ventilated" regions from segmentation of hyperpolarized gas images / total lung volume from segmentation of ^1^H images). In addition to calculation of VV%, images were qualitatively assessed for size and number of ventilation defects (regions of unventilated signal, defined as falling below 2 standard deviations of the noise) and signal heterogeneity in ventilated areas. Hyperpolarized gas and ^1^H pulse sequence parameters are listed in Table 2. In each case, a dose of ^3^He (99.25% of 100% ^3^He isotope, 0.75% N_2_) or 129‐enriched xenon (86% ^129^Xe) gas was balanced to 1 L with N_2_ in a Tedlar bag and inhaled via a sterilized air filter. Prior to image acquisition, subjects inhaled from functional residual capacity (FRC) after a period of steady breathing. Target inhaled gas doses were achieved to within a tolerance of ± 10 mL for ^3^He and ± 20 mL for ^129^Xe. Images with a signal‐to‐noise ratio (SNR) of <5 were not included in quantitative analysis. This threshold was determined by quantitative evaluation of the effect of SNR on ventilation heterogeneity^30^ by incrementally adding noise to high SNR ventilation images.

**Table 2:** Summary of MR Pulse Sequence Acquisition Parameters

Metric	^3^He VV%		^129^Xe VV%		^3^He ADC	^129^Xe ADC
Pulse sequence	^3^He 3D SSFP	^1^H 3D SPGR	^129^Xe 3D SSFP	^1^H 3D SPGR	DW 2D SPGR	DW 2D SPGR
FOV (cm)	∼40	∼40	∼40	∼40	∼40	∼40
Phase FOV	0.8	1.0	0.8	1.0	0.75	0.75
Matrix	100 × 80	100 × 100	100 × 80	100 × 100	64 × 64	64 × 48
Pixel size (mm)	4 × 4	4 × 4	4 × 4	4 × 4	6.25 × 6.25	6.25 × 8.33
# of slices	∼48	∼48	∼24	∼24	5	4
Slice thickness (mm)	5	5	10	10	15 (10 mm gap)	15 (10 mm gap)
Flip angle (°)	9	5	9/10^a^	5	4.8	6.7
TE/TR (msec)	0.6/1.9	0.6/1.9	2.2/6.7	0.6/1.9	4.8/10.0	12.5/27.0
BW(kHz)	±83.3	±83.3	±8	±83.3	±31.25	±2
Gas dose (ml)	200	N/A	600	N/A	300	600
Breath‐hold (s)	5	4	14	4	16	16
	∼14 (^3^He+gap+^1^H)		—		—	—
Δ (ms)	—		—		1.6 (τ=0.3;	5.0 (τ=0.3;
	—		—		δ=1.0; X=0)	δ=3.0; X=1.4)
N_D_	—		—		6	4
*G*_m_ (mT.m^−1^)	—		—		31.6	32.2
*b*_1,2_ (s.cm^−2^)	—		—		0; 1.6	0; 8.0

SSFP: steady‐state free precession; SPGR: spoiled gradient echo; FOV: field of view; TE: echo time; TR: repetition time; BW: bandwidth; Δ: diffusion time, τ: ramp time, δ: plateau time; X: separation of gradient lobes; N_D_: number of diffusion‐weighted interleaves; G_m_: maximum gradient amplitude; *b*_1,2_: *b* values of first two interleaves.

FOV and ventilation # of slices were increased if necessary to cover the whole lungs of larger patients.

a Flip angle was 9 ° for COPD patients, 10 ° for NSCLC patients.

Healthy nonsmokers (group a), ex‐smokers, and COPD patients underwent 2D multislice ^3^He and ^129^Xe diffusion‐weighted lung MRI to acquire ADC maps of the lungs. ADC values were calculated from the first two interleaves of a multiple *b*‐value 2D diffusion‐weighted spoiled gradient echo sequence. Acquisition parameters and bipolar diffusion gradient timing parameters are summarized in Table 2. Gradients were designed to achieve comparable diffusion weighting for the two nuclei, and slice locations were matched (with an additional anterior slice for ^3^He acquisitions). The same SNR threshold as ventilation imaging was applied to diffusion‐weighted MRI data. Note: Healthy nonsmokers (group a) and ex‐smokers did not undergo ventilation imaging in this study. Likewise, patients with NSCLC and healthy group b did not undergo diffusion‐weighted MRI.

Patients with COPD underwent the complete MR protocol on four separate scan sessions in total: twice on day 1; once on day 2; and once 2 weeks after day 1, to assess the repeatability of ^3^He and ^129^Xe metrics.

### Pulmonary Function Testing

PFTs, including the diffusing capacity of the lung for carbon monoxide (D_LCO_) and spirometry, were performed by each COPD patient, once on the same day as each MR session (three times in total, always following MRI). Healthy nonsmokers and ex‐smokers performed the same tests immediately after their single‐timepoint diffusion‐weighted MR scans, and NSCLC patients performed PFTs within ±1 week of MRI.

### Statistical Analysis

VV% and global mean ADC (ADC_glob_) values derived from ^3^He and ^129^Xe MRI were compared for differences in each metric between two, or more than two, *subject groups* with conventional *t*‐tests or analyses of variance (ANOVA), respectively. Differences in metrics derived between *gas nuclei* were analyzed using Wilcoxon signed rank tests. Spearman's rank correlation coefficients were derived to quantify the relationship between ^3^He and ^129^Xe datasets, and Bland–Altman analysis was used to visualize systematic differences in VV% between the two nuclei. MRI metrics were compared and correlated against PFT results by calculating Spearman's rank coefficients. The mean of repeated measurements from each COPD patient was used to represent a single datapoint for the above statistical tests.

Where repeatability data were available, the coefficient of variation (CV) of each parameter was calculated as the ratio of the standard deviation (SD) to the mean over all repeated measurements, expressed as a percentage. In addition, two‐way mixed intraclass correlation (ICC) coefficients were calculated for absolute agreement between repeat measurements. Statistical analyses were performed using IBM SPSS Statistics (v. 23, Armonk, NY) and R (R3.4, R Foundation for Statistical Computing, Vienna, Austria), and statistical significance level was set to *P* < 0.05.

## Results

### Imaging Data Quality

From the healthy nonsmokers cohort, ^129^Xe diffusion‐weighted MR images from one subject exhibited insufficient SNR for accurate calculation of ADC maps. From the COPD cohort, one ^3^He and one ^129^Xe ADC dataset, and two ^3^He and two ^129^Xe VV% datasets (each out of a total of 20 datasets for each nucleus, each metric) were either not successfully acquired or image SNR was unsatisfactory. From the NSCLC patient cohort, one ^3^He dataset and one ^1^H dataset was not acquired successfully, and two ^129^Xe datasets showed low SNR.

All other recorded data were of sufficient SNR for analysis: ie, for ^3^He, >95% of ADC data and >90% of VV% data were acceptable; for ^129^Xe, 95% of ADC data and >85% of VV% data were acceptable. All gas doses were well‐tolerated and no significant side effects or adverse events were reported. Mean MR parameters and PFT results are shown in Table 1.

### Ventilation Imaging

Representative ^3^He and ^129^Xe coronal ventilation images and corresponding structural ^1^H images from patients with NSCLC and COPD are shown in Fig. 1 (left), alongside images from a healthy nonsmoker. Considerable ventilation abnormalities were observed in patients with NSCLC, with most patients exhibiting a heterogeneous distribution of ventilated airspaces and complete absences of ventilation in the region of the lung associated with malignancies (as identified on structural ^1^H MRI). Severe ventilation defects were detected in most COPD patients, and entire lobes of the lungs were often observed to be completely unventilated.

**Figure 2:**
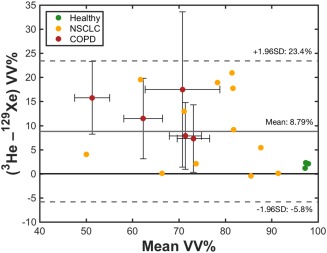
Bland–Altman analysis of ^3^He and ^129^Xe VV% values in patients with NSCLC and COPD and healthy nonsmokers group b. The solid gray line indicates the mean difference (bias) between ^3^He and ^129^Xe VV%, and dashed lines represent ±1.96 standard deviations from the mean. Datapoints and error bars for COPD patients denote intrasubject means and standard deviations of repeated acquisitions, respectively.

By pooling data from healthy nonsmokers (group b) and patients with NSCLC and COPD, a significant positive correlation between ^3^He and ^129^Xe VV%, was identified (Spearman's correlation coefficient, *r* = 0.860, *P* < 0.001). Derived VV% values were larger on average for ^3^He when compared with ^129^Xe (mean over all healthy group b, NSCLC, and COPD subjects); *P* < 0.001 (Wilcoxon signed rank) and qualitatively, typically larger and more numerous ventilation defects could be observed in ^129^Xe images. A Bland–Altman plot of the systematic differences between ^3^He and ^129^Xe VV% is shown in Fig. 2, indicating a bias of +8.79% towards higher ^3^He VV%. Analyzing data from COPD and NSCLC patients separately, intragroup correlations between ^3^He and ^129^Xe VV% were statistically significant for NSCLC patients (*r* = 0.657, *P* = 0.024) but not COPD patients (*r* = 0.700, *P* = 0.233). Additionally, ^3^He VV% values were found to be higher than ^129^Xe VV% values when COPD (*P* = 0.063, close to significant) and NSCLC (*P* = 0.002, significant) patient data were analyzed separately. There were no significant differences in either mean ^3^He VV% (*P* = 0.095) or mean ^129^Xe VV% (*P* = 0.085) values between the two patient groups, although the VV% trended to be lower in COPD patients compared with NSCLC patients.

### Diffusion‐Weighted Imaging

**Figure 3:**
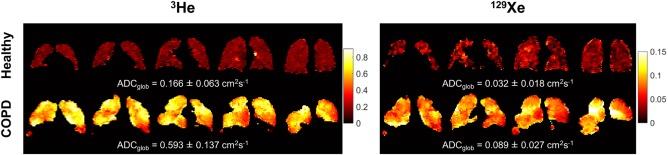
Representative dataset of ^3^He and ^129^Xe ADC maps obtained from a healthy nonsmoker (group a) and a patient with COPD. The mean ADC over all slices is quoted underneath each dataset.

Examples of ^3^He and ^129^Xe ADC maps obtained from a healthy nonsmoker and a patient with COPD are shown in Fig. 3. ADC_glob_ values of both nuclei were significantly elevated in COPD patients when compared to both healthy nonsmokers (group a) and ex‐smokers: ^3^He ADC_glob_ = 0.432 ± 0.127 cm^2^.s^−1^ in COPD patients, 0.211 ± 0.022 cm^2^.s^−1^ in ex‐smokers (*P* = 0.036 vs. COPD patients) and 0.190 ± 0.017 cm^2^.s^−1^ in healthy nonsmokers (*P* = 0.028 vs. COPD patients); mean ^129^Xe ADC_glob_ = 0.073 ± 0.019 cm^2^.s^−1^ in COPD patients, 0.042 ± 0.004 cm^2^.s^−1^ in ex‐smokers (*P* = 0.045 vs. COPD patients) and 0.038 ± 0.003 cm^2^.s^−1^ in healthy nonsmokers (*P* = 0.029 vs. COPD patients). Ex‐smoker and healthy nonsmoker ADC_glob_ values were statistically indistinguishable; *P* = 0.214 and *P* = 0.114 for ^3^He and ^129^Xe data, respectively.

**Figure 4:**
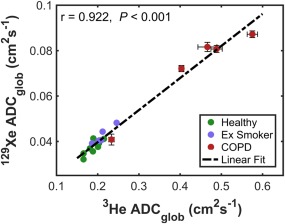
Correlation between mean ^3^He and ^129^Xe ADC_glob_ in healthy nonsmokers (group a), ex‐smokers, and patients with COPD, with associated Spearman's correlation coefficient (*r*) and *P* value of statistical significance. The dashed line represents a linear fit to the data and error bars represent the standard deviation of repeated scans for each COPD patient.

A strong positive correlation between ^3^He and ^129^Xe ADC_glob_ was found when pooling data from healthy nonsmokers group a, ex‐smokers and COPD patients together (Fig. 4: Spearman's *r* = 0.922, *P* < 0.001). Analyzing the three subject groups separately, significant correlations between ^3^He and ^129^Xe ADC_glob_ were observed for each of the three groups (*P* < 0.05).

### MR Metrics vs. PFTs

A summary of the statistical analyses of relationships between MR metrics and PFTs is presented in Table 3. Both ^3^He and ^129^Xe VV% exhibited significant correlations with FEV_1_ and FEV_1_/FVC at *P* < 0.001 (except ^129^Xe VV% vs. FEV_1_, *P* < 0.05 significance level). Correlations of VV% values derived from both nuclei with D_LCO_ were relatively weaker, although still significant at the *P* < 0.05 level. Note: One significant outlying datapoint from a patient with NSCLC with %‐predicted spirometry ∼90% but low VV% values, ∼50% for both nuclei, was excluded from the analysis presented in Table 3. When these outlying data were included in the analysis, the correlation statistics were severely affected; correlations of VV% with D_LCO_ were no longer significant (*P* > 0.05) and all other correlations were significant at the *P* < 0.05 level, rather than *P* < 0.001 (Table 3 caption).

**Table 3:** Correlations Between MRI‐Derived ^3^He and ^129^Xe VV% and ADC Measurements and PFTs

r (*P*)	^3^He ADC	^129^Xe ADC	^3^He VV%	^129^Xe VV%
D_LCO_ (%‐pred)	−0.537 (0.018^a^)	−0.628 (0.005^a^)	0.524^c^ (0.019^a^)	0.476^c^ (0.035^a^)
FEV_1_ (%‐pred)	(−0.724 <0.001^b^)	−0.834 (<0.001^b^)	0.732 (<0.001^b^)	0.648 (0.002^a^)
FEV_1_/FVC	−0.747 (<0.001^b^)	−0.672 (0.001^a^)	0.819 (<0.001^b^)	0.744 (<0.001^b^)

a Correlations at the statistical significance level of *P* < 0.05.

b Correlations at the statistical significance level of *P* < 0.001.

c Results of correlating VV% values with PFTs represent the statistics obtained when outlying data associated with one NSCLC patient was excluded. When this data‐point was included, the correlation coefficients (*P* values) for VV% altered as follows: ^3^He, 3 rows: 0.432 (0.052); 0.592 (0.005^a^); 0.559 (0.012^a^); ^129^Xe, 3 rows: 0.387 (0.084); 0.531 (0.012^a^); 0.529 (0.015^a^).

%‐pred: %‐predicted pulmonary function test result.

ADC_glob_ values also showed strong correlations with PFTs; notably, correlations of ^3^He ADC_glob_ with both FEV_1_ and FEV_1_/FVC were significant at the *P* < 0.001 level, while correlations with D_LCO_ were significant at the *P* < 0.05 level (Table 3). Similarly, correlations of ^129^Xe ADC_glob_ with FEV_1_ were significant to *P* < 0.001, and correlations with FEV_1_/FVC and D_LCO_ were significant to *P* < 0.05.

### Repeatability

**Figure 5:**
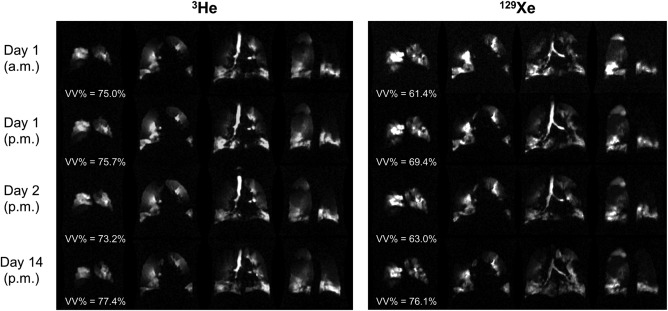
Selected ^3^He and ^129^Xe ventilation image slices acquired at each of the four scan timepoints of the repeatability study from a COPD patient. Calculated ventilated volume percentages (VV%) are quoted underneath each respective set of images.

In COPD patients, both ^3^He and ^129^Xe ventilation images appeared qualitatively similar (in terms of visual ventilation defect prevalence and ventilation homogeneity) at each of the four scan timepoints, as shown for a representative example in Fig. 5. Mean CV values of ^3^He and ^129^Xe VV% between all repeated scans were 4.08% and 13.01%, respectively (Table 4). According to ICC analysis, both ^3^He VV% (ICC coefficient 0.541, *P* = 0.061) and ^129^Xe VV% (coefficient 0.458, *P* = 0.095) repeatability—calculated by considering data from all scan timepoints—was classified as just beyond the 95% confidence level. The CV of ^3^He VV% was comparable to that of PFTs, while the ^129^Xe VV% CV was higher.

**Table 4:** Repeatability of PFTs and MRI Metrics in Patients With COPD

	ICC (*P* value)	CV mean (%)	CV range (%)
FEV_1_ (%‐pred)	0.934 (<0.001^a^)	4.22	2.16–6.35
FEV_1_/FVC	0.987 (<0.001^a^)	2.34	1.49–3.80
D_LCO_ (%‐pred)	0.992 (<0.001^a^)	8.73	3.91–16.15
^3^He VV%	0.541 (0.061)	4.08	2.21–8.86
^129^Xe VV%	0.458 (0.095)	13.01	9.62–25.28
^3^He ADC_glob_	0.995 (<0.001^a^)	2.98	1.48–5.61
^129^Xe ADC_glob_	0.936 (<0.001^a^)	2.77	1.68–5.88

ICC = intraclass correlation coefficient; CV = coefficient of variation.

Statistical significance level of *P* < 0.05.

a Statistical significance level of *P* < 0.001.

**Figure 6:**
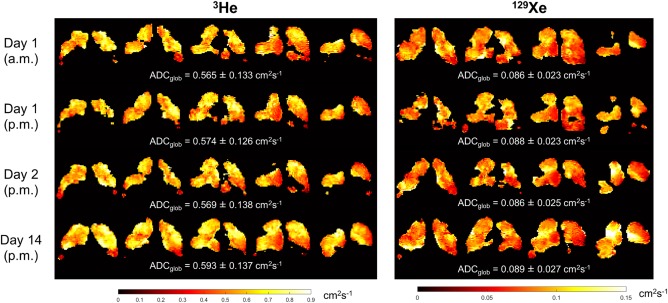
Representative ^3^He and ^129^Xe ADC maps acquired at each of the four scan timepoints of the repeatability study from a COPD patient. Global mean ADC values (ADC_glob_) are quoted underneath each respective set of maps.

Good agreement in the appearance of both ^3^He and ^129^Xe ADC maps was observed for each scan timepoint, as illustrated in Fig. 6. ^3^He and ^129^Xe ADC_glob_ values were highly repeatable over all scan sessions, with ICC coefficients for interscan agreement (0.995 and 0.936 for ^3^He and ^129^Xe, respectively) significant at the *P* < 0.001 level, and mean CV values of <3% in both cases (see Table 4). ICC coefficients and CVs of ADC_glob_ values were comparable to those of FEV_1_ and FEV_1_/FVC, and considerably less than those of D_LCO_ and VV%.

## Discussion

This work indicates that ^129^Xe lung MRI can provide equivalent regional information on lung ventilation and microstructure to ^3^He MRI at to‐date the most‐reported field strength for lung MRI, 1.5T. Specifically, we demonstrated a clear agreement between ^129^Xe ventilation and diffusion MRI measurements and ^3^He equivalent measurements; these findings substantiate previous work from the Robarts group that was performed at 3T with lower spatial resolution imaging.^3^, ^15^, ^16^ We also quantified the repeatability of ^129^Xe ventilation and diffusion‐weighted MRI metrics against ^3^He equivalent measurements in COPD patients.

^3^He and ^129^Xe VV% correlated strongly, with a positive bias towards increased VV% for ^3^He. Discrepancies can likely be attributed to differences in image slice thickness, gas polarization, and the fundamental properties of the gases as discussed below. The generally lower VV% of ^129^Xe may be explained by the ∼5‐fold lower diffusion coefficient of xenon in air (as confirmed by the mean ADCs of the two gases measured in healthy lungs here), and the resulting slower penetration of partially obstructed airways, although the relatively lower mean SNR of ^129^Xe ventilation images may also contribute to this observation. In patients with severe disease, partially obstructed airways may cause an accentuation of this diffusion‐related effect, leading to observation of further ventilation abnormalities with ^129^Xe when compared with ^3^He. We note that a time‐dependent diffusive effect of delayed filling of ventilation defects and collateral ventilation has been observed in COPD patients by ^3^He MRI,^31^ but the equivalent measurements has not yet been reported for ^129^Xe. A reduced VV% for ^129^Xe when compared with ^3^He was also reported previously at 3T^3^, ^16^ and attributed to lower diffusivity. Furthermore, systematically higher absolute ventilated volumes have been reported for ^3^He when compared with ^129^Xe at 3T,^15^ wherein it was suggested that ^3^He has a higher propensity to flow through collateral channels in COPD patients. Nevertheless, this property of ^129^Xe could prove useful in the assessment of early (ie, small‐scale) structural abnormalities of distal airways and lung parenchyma in a variety of pulmonary diseases, provided that equivalent image SNR and spatial resolution can be achieved as that of ^3^He MRI.

The lower CV values (and higher ICC coefficients) of ^3^He VV% when compared with ^129^Xe VV% are likely attributable to the generally lower and more variable SNR of ^129^Xe images acquired at the time of the study (mean ± standard deviation of SNR over all NSCLC and COPD patients: 23.6 ± 16.8 for ^129^Xe; 30.6 ± 10.8 for ^3^He), and the differences in lung inflation level in some cases between ^129^Xe and anatomical ^1^H images acquired in a separate breath‐hold.^28^ As mentioned in the Materials and Methods, polarization levels, and thus image SNR, affect the ventilation heterogeneity and therefore images with low SNR (<5) can significantly distort the physiological interpretation of the data. The relatively lower SNR may be explained by the fact that a prototype ^129^Xe polarizer (producing ^129^Xe polarization ∼10–15%) was used for most ^129^Xe experiments in this work.^12^ We have since developed and optimized a clinical‐scale polarizer capable of rapidly producing ^129^Xe polarized to ∼40%, which will enable improved ^129^Xe image SNR in future studies.^27^ Nevertheless, the CV of ^129^Xe VV% is severely biased by an outlying subject (CV of 25%); the CV in all other COPD patients was ∼10%. The ^129^Xe images from the first scan session in this outlying subject were excluded from analysis because of insufficient SNR. Despite this observation, no other metrics showed abnormally high CV for that patient and no differences in the patient's clinical status were noted.

The high regional heterogeneity of COPD may explain the increased CV of VV% when compared with ADC_glob_ (ADC maps are of lower resolution than ventilation images), although we note that, qualitatively, the prominent areas of ventilation defect were observed repeatedly over scan sessions. Furthermore, the variability of bronchodilator therapy applied on a day‐to‐day basis will potentially impact VV% more severely than ADC. Nonetheless, both ^3^He and ^129^Xe VV% CV values calculated from COPD patient data are similar to week‐to‐week percentage variability in FEV_1_ and D_LCO_,^32^ and same‐day ^3^He VV% variability in pediatric CF patients,^21^ as reported earlier, although poorer than or comparable to the repeatability of PFTs measured in the present work. Our ICC values for ^129^Xe VV% are considerably lower than same‐session (10 minutes apart) ^129^Xe VDP ICCs reported by Ebner et al^23^; however, the differences can potentially be explained by the fact that in that work, patients did not leave the scanner between scans, no multisession/multiday data were recorded, and the number of patients was significantly higher than in the present work. Otherwise, the results of ^129^Xe VV% and ADC repeatability have, to our knowledge, not been previously reported, and hence our measurements can only be assessed against literature data for equivalent ^3^He measurements. Resolution of some of the issues discussed herein, including utilization of the higher performance polarizer, should lead to improvements in the repeatability of ^129^Xe VV% to a similar level as ^3^He, thus facilitating translation to large‐scale clinical studies.

Typically, lower imaging bandwidths and larger diffusion gradients are required for ^129^Xe when compared with ^3^He lung MRI, necessitating longer breath‐hold times or lower spatial resolutions. Additionally, until recently, ^129^Xe polarizer performance has not been adequate to overcome the inherent MR signal disadvantages compared with ^3^He. As such, a compromise was reached in this work, such that the in‐plane resolution of ^129^Xe ventilation imaging acquisitions matched that of ^3^He, but the slice resolution was halved. Developments in compressed sensing (CS) and parallel imaging acquisition strategies for accelerated imaging^33^, ^34^ should help permit equivalent, isotropic resolution ^129^Xe, and ^3^He ventilation imaging within a clinically feasible breath‐hold in the near future, and facilitate same‐breath ^129^Xe and ^1^H imaging for improved ^129^Xe ventilation volumetry. Moreover, it has recently been demonstrated that 3D multiple *b*‐value diffusion‐weighted CS‐based acquisition strategies for whole‐lung morphometric analysis developed for diffusion‐weighted ^3^He MRI^35^ are readily transferable to ^129^Xe.^36^ Although the through‐plane ^129^Xe ventilation and diffusion image resolution in this work is already superior to previous reports at 3T, the adoption of CS methods for both applications should enable these resolution limits to be pushed further towards those attainable with ^3^He.

Mean ADC_glob_ values of both nuclei exhibited excellent correlation with each other and high repeatability in COPD patients, with comparable or lower interscan CVs than conventional PFTs, suggesting that ^129^Xe diffusion‐weighted MR has approximately equal sensitivity to ^3^He for quantitative assessment of lung microstructural changes, and sufficient reliability for routine clinical application.

The observation of elevated ADC_glob_ in COPD patients agrees with previous reports for both ^3^He^3^, ^26^ and ^129^Xe,^6^, ^7^ and has been well characterized. Similarly, increased ADC_glob_ in subjects with smoking history is consistent with previous observations,^7^ although the ^129^Xe *b*‐values used in those works differ from this work. As a consequence of the non‐Gaussian phase dispersion of the ^3^He and ^129^Xe diffusion regime experienced in the lungs, ADC_glob_ exhibits some variation with *b*‐value,^37^ which constrains the validity of interstudy comparisons. As such, we recommend that for ease of implementation and comparison across sites, a standardized *b*‐value and diffusion time should be adopted for ^129^Xe diffusion‐weighted MRI, similar to the value of 1.6 s.cm^−2^ now widely used for ^3^He.

CV values of both ^3^He and ^129^Xe ADC_glob_ are less than those of previously reported same‐day variability in FEV_1_,^32^ and substantially lower than measured variability in D_LCO_ (∼9%)^38^. D_LCO_ is perhaps the most relevant PFT to compare with ADC, since both measurements rely on gas diffusion within the alveoli, albeit for studying different lung physiology. Thus, the observed repeatability is extremely promising for advancing ^129^Xe diffusion‐weighted MRI to large‐scale multisite trials with the goal of routine clinical implementation. Additionally, our reported CV values of both ^3^He and ^129^Xe ADC_glob_ are less than ^3^He ADC CVs reported for healthy subjects and patients with emphysema at 1.5T,^26^ and are comparable to ^3^He CVs measured at 3T.^20^

The design of the present study has some limitations that could be effectively resolved in future studies. Although a reasonable number of patients were included in this study (37 in total), repeatability experiments were only performed on five patients and, thus, a follow‐up comprehensive, repeatability‐specific study is required with an increased number of subjects, including both patients and volunteers. In such a study, it may be statistically meaningful to distinguish same‐day from 2‐week repeatability, and therefore better identify potential sources of physiological variability between different scan sessions. Due to a few missing datasets, the current data is insufficient in quantity for such statistically sound intertimepoint repeatability analysis; hence, the repeatability was only analyzed over all scan sessions. Data regarding day‐to‐day and week‐to‐week repeatability is critical for understanding the natural physiological variability in VV% and ADC_glob_ in patients and discriminating this from measurement uncertainty.

In conclusion, the findings reported herein demonstrate that ^129^Xe is approaching readiness as a clinically viable alternative to ^3^He for quantitative lung ventilation and microstructural MRI studies. For future dissemination and multisite cross‐platform clinical trials, the fact that our results agree with many of the trends seen in prior studies that benchmarked ^3^He and ^129^Xe MRI measurements at 3T is encouraging. Although quantitative measurements of ventilation and ADC will be biased by magnetic susceptibility effects at the two field strengths, we believe that equivalent functional information can be achieved with both gases at both field strengths. In addition, the high repeatability of both ^3^He and ^129^Xe ADC observed here is perhaps the most convincing evidence to date that ^129^Xe ADC imaging has developed into a robust microstructural imaging methodology. Moreover, this report builds upon previous measurements at 3T in terms of improved spatial resolution of ^129^Xe imaging, and the realization of 3D DW‐MRI with both nuclei^35^, ^36^ is extremely promising for further validation and clinical application in the near future. The preliminary assessment of the repeatability of ^129^Xe ventilation and diffusion MRI biomarkers addresses a gap in knowledge that was not considered prior to this work and paves the way for further, more comprehensive multisite repeatability studies.

## Conflict of Interest

Dr. Yates was involved in the study design and imaging protocol for the COPD imaging metric repeatability study. She is part of the Novartis imaging biomarker team and this work was not related to a clinical trial of a therapeutic.
